# In vivo bactericidal efficacy of the Ti6Al4V surface after ultraviolet C treatment

**DOI:** 10.1007/s10195-016-0407-x

**Published:** 2016-05-02

**Authors:** Juan A. Constantino, María Delgado-Rastrollo, Miguel A. Pacha-Olivenza, M. Luisa González-Martín, Manuel Quiles, C. Pérez-Giraldo, José M. Bruque, Amparo M. Gallardo-Moreno

**Affiliations:** 1 Orthopaedic and Traumatic Service, Complejo Hospitalario Universitario de Badajoz, Avenida Tellez Lafuente s/n, 06010 Badajoz, Spain; 2 Department of Biomedical Sciences, Microbiology Area, Faculty of Medicine, University of Extremadura, Avda de Elvas s/n, 06006 Badajoz, Spain; 3 Department of Applied Physics, Faculty of Science, University of Extremadura, Avda de Elvas s/n, 06006 Badajoz, Spain; 4 Networking Research Center on Bioengineering, Biomaterials and Nanomedicine (CIBER-BBN), Badajoz, Spain

**Keywords:** Ti6Al4V, Ultraviolet, Bactericidal, Infection, In vivo

## Abstract

**Background:**

Biomaterial-associated infections are one of the most important complications in orthopedic surgery. The main goal of this study was to demonstrate the in vivo bactericidal effect of ultraviolet (UV) irradiation on Ti6Al4V surfaces.

**Materials and methods:**

An experimental model of device-related infections was developed by direct inoculation of *Staphylococcus aureus* into the canal of both femurs of 34 rats. A UV-irradiated Ti6Al4V pin was press-fit into the canal by retrograde insertion in one femur and the control pin was inserted into the contralateral femur. To assess the efficacy of UV radiation, the mean colony counts after inoculation in the experimental subjects and the control group were compared at different times of sacrifice and at different inoculum doses.

**Results:**

At 72 h, the mean colony counts after inoculation in experimental femurs were significantly lower than those of the control group, with a reduction percentage of 76 % (*p* = 0.041). A similar difference between control and experimental pins was observed at 24 h using an inoculum dose <10^4^ colony-forming units (CFU), for which the reduction percentage was 70.48 % (*p* = 0.017).

**Conclusion:**

The irradiated surface of Ti6Al4V is able to reduce early bacterial colonization of Ti6AlV pins located in the medullar channel and in the surrounding femur. The reductions depend on the initial inoculums used to cause infection in the animals and the greatest effects are detected for inoculums <10^4^ CFU.

**Level of evidence:**

Not applicable.

## Introduction

The global market of biomaterials was estimated at US$ 150–200 billion in 2012 including all diagnostic and therapeutic equipment, and the largest market for biomaterial-based products is orthopedic biomaterials followed by cardiovascular and drug delivery materials [1]. In fact, bone and joint degenerative and inflammatory problems account for half of all chronic diseases in people >50 years of age in developed countries. These diseases often require surgery, including total joint replacement in case of deterioration of the natural joint [2].

One of the most important complications related to fracture fixation and osseous substitution is the appearance of infectious processes on the surface of the implanted biomaterials, which bring about multiple complications such as long hospitalization stays, complex revision processes, implant looseness and failure, the high cost of replacement surgery, and subsequent patient pain [3–6]. Bacterial colonization and further biofilm formation on orthopedic devices are the preludes to such device-related infections, and the most standardized strategy to fight against these episodes is antibiotic prophylaxis before the appearance of the infection. A mimimum 6-week course of systemic antibiotics is needed to treat infections that involve bone [6]. This continuous use of antibiotics has caused an increase in the number of multi-resistant bacteria such as methicillin-resistant *Staphylococcus aureus* [7], which is the most frequently isolated pathogen in implant-related infections. Several authors have already pointed out that systemic administration of antibiotics, despite being a useful intervention, may not be successful in all patients [8, 9], and it is necessary to find new ways to minimize the incidence of infections.

Titanium alloys, such as Ti6Al4V, are widely used in orthopedic and dental applications due to their low density, excellent mechanical and anti-corrosive properties and good biocompatibility [10]. One of the reasons for these beneficial properties is the thin passivation layer which spontaneously forms on the surface of the alloy, predominantly composed of amorphous or poorly recrystallized titanium dioxide (TiO_2_) [11, 12]. The inert TiO_2_ layer minimizes ion release from the implant to the surrounding tissues and hence inflammatory reactions in the body [13]. In addition, a singular property of TiO_2_ is its semiconductor character, with a band gap of approximately 3.2 eV, which means that the oxide can be excited under illumination with wavelengths within the ultraviolet (UV) spectra. It has been widely reported that illumination of TiO_2_ with UV light produces hydrophilization of its surface [14, 15], and is able to induce photocatalytic surface reactions under excitation. TiO_2_ photocatalysis acquires strong oxidizing power and can decompose various microorganisms and organic compounds by generating active oxygen species [16, 17]. For this reason, industrial applications of anti-bacterial TiO_2_-coated surfaces can be found in different areas such as water disinfection, air purification or food surface preparation [17–21].

Nevertheless, although there is no doubt about the bactericidal effect of TiO_2_-coated biomaterials upon UV irradiation [22, 23], there have been very few reports about its application on implant-related materials, and these studies have been mainly carried out with titanium-based materials not fully placed inside the body. For example, Choi et al. worked with orthodontic materials under UV irradiation by employing selected strains of *Lactobacillus acidophilus* and *Streptococcus mutans* [24, 25]. Oka et al. demonstrated the in vivo bactericidal ability of UV-A light on percutaneous implants used in external fixation by directly irradiating the external part of the pins for 60 min daily [26].

Despite these interesting studies, the impossibility of applying the well-known antibacterial properties of TiO_2_-coated surfaces while they are directly exposed to an energetic UV source to the medicine field, led our group to investigate the characteristics of the irradiated surface after, and not during, exposure to a UV source.

Our research group has recently studied in vitro the remaining antibacterial effect that the surface of Ti6Al4V exhibits after being illuminated by UV-C light [27, 28]. The work was carried out with different strains of two clinical isolates—*S. epidermidis* and *S. aureus*—on the bacterial adhesion process and the viability of adhered bacteria. Our experiments showed that the irradiated material diminished not only bacterial adhesion but also bacterial retention ability. In addition, the irradiated surface compromised the viability of the adhered bacteria within the first 2 h after turning off the UV source. This bactericidal effect, checked not only for Gram-positive bacteria but also for Gram-negative cells, could bring important benefits to the implantation field since bacteria introduced accidentally during surgery adhere immediately to the implant surface.

Nevertheless, despite the usefulness of the previous research, the in vivo scenario can differ from the in vitro situation, and this must be taken into account when proposing UV irradiation as an antimicrobial method for titanium implants. For this reason, the aim of the present study is to check whether the in vitro antimicrobial properties exhibited by Ti6Al4V are maintained under in vivo conditions at the initial stages of biofilm formation. To this end, irradiated and non-irradiated implants were inserted in the medullar channel of rat femurs and their bacterial colonization evaluated at 72 h. Femurs were also analyzed to check whether there was any bactericidal effect on the material surrounding the implant.

## Materials and methods

### Ti6Al4V

**Fig. 1:**
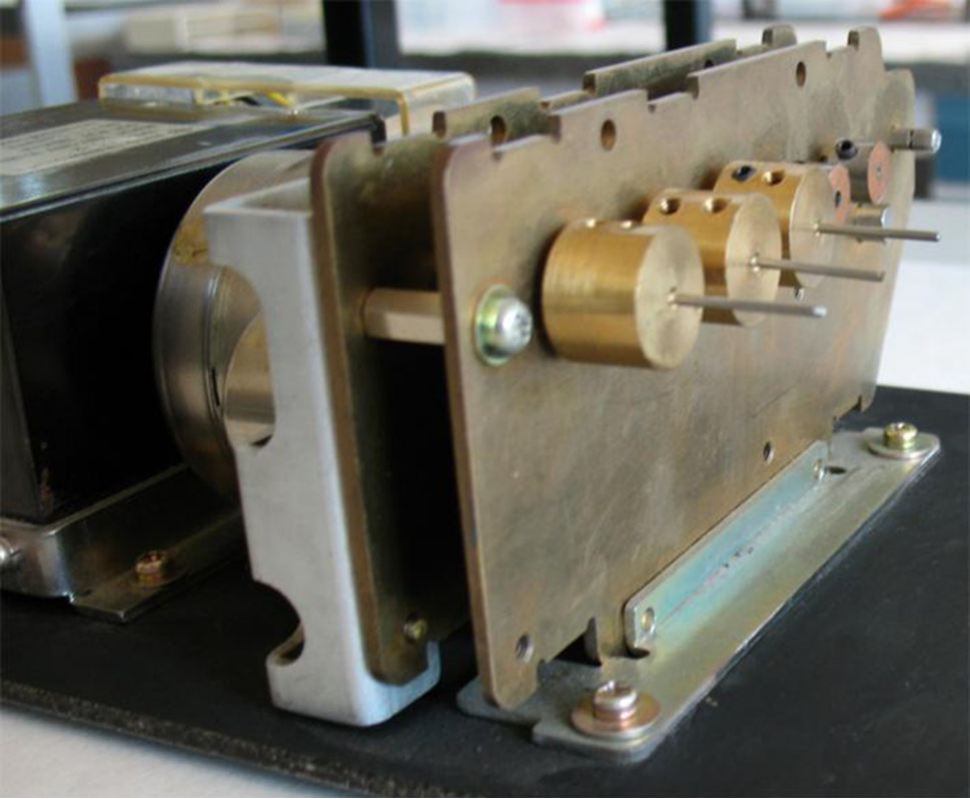
Homemade experimental design to irradiate three pins simultaneously

Ti6Al4V wires (1.2 × 150 mm) were supplied by Kirschner Maschinenbau GmbH (Unterschneidheim, Germany). These were cut into 20-mm lengths and the edges were polished (we will refer to these pieces as pins or implants). The roughness of the surface was Ra = 0.95 ± 0.05 μm, measured with a stylus profilometer (TR200; Qualitest, Canada). Prior to use, all pins were carefully cleaned with DSF disinfectant (DERQUIM DSF 11; Panreac Quimica S.A., Spain) at 60 °C by vigorously rubbing with a smooth cotton cloth, then rinsed repeatedly with distilled water and sonicated in deionized water (Milli-Q system), 70 % acetone and finally ethanol for periods of 10 min each. They were dried in an oven at 40 °C for 1 h and stored in a desiccator for 24 h. [28]. Samples used as controls were not subjected to any further treatment. A second set of samples was exposed to a UV-C source for different times until complete hydrophilization of the surface was reached. G15-T8 UV lamps, emitting predominantly at a wavelength of 254 nm were kindly provided by Philips Iberica, Spain. The samples were placed in a homemade rotatory system to ensure full irradiation of the curved surface (Fig. 1).

They were placed 10 cm from the UV light source and received an intensity of around 4.2 mW cm^−2^. The possibility of irradiating three pins simultaneously allowed better comparisons between triplicate experiments. The irradiation installation was inside an opaque chamber to prevent interference from roomlight or daylight or harm to users. This irradiation study was conducted prior to the experiments on animals. Samples were irradiated for periods of 15, 24, 36, 48 and 72 h with the aim of finding an irradiation time that guaranteed complete hydrophilization of the Ti6Al4V surface. This specific irradiation time would be selected for all subsequent experiments on rats.

### Hydrophobic/hydrophilic characterization

The samples before and after irradiation were subjected to hydrophobic analysis in order to check whether the irradiation time was able to induce hydrophilic changes on the surface of the pins similar to those observed in previous studies with Ti6Al4V disks [27]. After irradiation, samples were horizontally suspended inside the environmental chamber of a Goniometer (G211; Krüss, Hamburg, Germany) with the help of two holders located at both ends of the sample. Water (Milli-Q Plus) droplets were carefully deposited on the top part of the surface of the pins using the automatic deposition protocol of the device. Different droplet volumes were tested according to the small curved area of the contact surface.

The volume that allowed stable contact angles immediately after deposition of the drop onto the surface of the pins was 1 μl. The number of pins used for each irradiation time was three and the total number of droplets for each time varied between six and nine depending on the characteristics of the surfaces (more or less hydrophilic). In summary, the total number of droplets on non-irradiated pins was 9 (three droplets for each pin) and 38 on UV-C irradiated pins.

### Bacterial strains and culture

*S. aureus* ATCC29213 was stored at −80 °C in porous beads (Microbank; Pro-Lab Diagnostics, USA). From the frozen stock, blood agar plates were inoculated and incubated at 37 °C to obtain cultures. A volume of 3 ml of Trypticase™ Soy Broth (BBL™; Becton–Dickinson and Company, Sparks, MD, USA) was inoculated with these cultures and incubated for 18–24 h at 37 °C. Bacteria were then harvested by centrifugation for 5 min at 3,000 rpm (Sorvall TC6; Dupont, USA) and washed three times with 0.15 M sterile phosphate-buffered saline (PBS, pH 7.2). Finally, bacteria were re-suspended in sterile PBS to a final transmittance of 82 % (equivalent in the McFarland standards to 1×10^8^ colony-forming units [CFU]/ml) and further diluted to obtain concentrations ranging from approximately 10^5^−10^7^ bacteria ml^−1^. A 5-μl volume of the different bacterial suspensions was used to inoculate the animals. The influence of size of inoculum in detecting differences between our specific surface treatment and the control surface was a main objective of this research and inoculum selection was based on previous studies (see ‘Discussion’ section).

### Animals and surgical technique

**Fig. 2:**
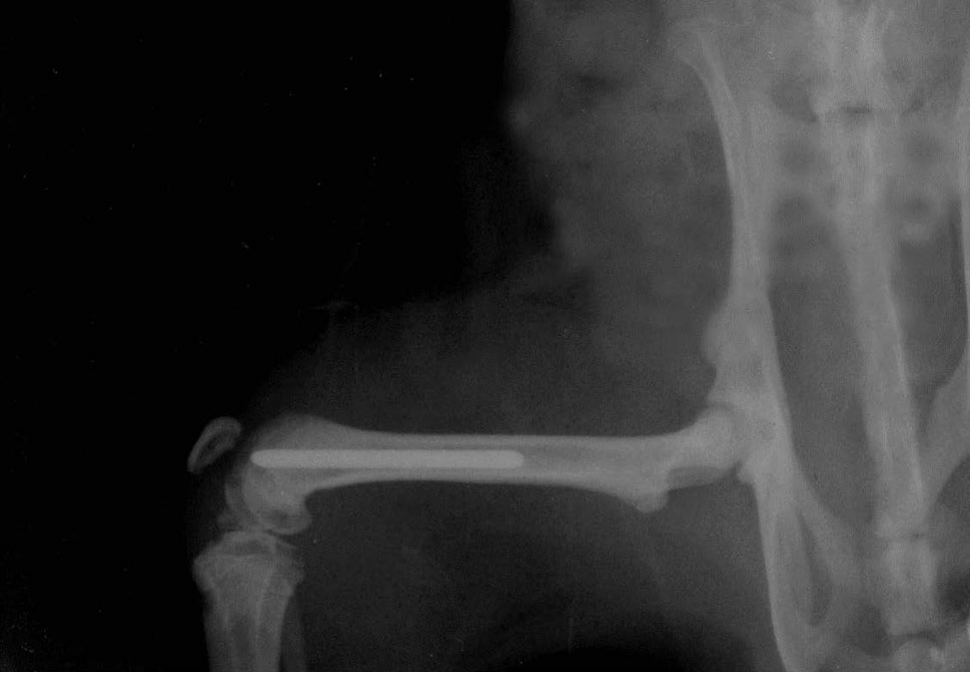
Pin implanted in rat femur. The surgical technique provided full pin insertion and isolation from the knee motion

All animal experiments were approved by the ethical board of the Extremadura University (file number 161/2009). Rules established in the National Research Council’s guide for animal experimentation were followed for animal handling and care. A total of 36 Wistar male rats with an average weight of 350 g were randomly assigned to three groups with different sacrifice times between 24 and 72 h and with different doses of inoculum between 10^3^ and 10^6^ CFU. The rats were individually caged, fed a standard diet, and provided with water ad libitum. They were anesthetized by intraperitoneal injection of 50 % ketamine, 40 % diazepam and 10 % atropine for surgery [4]. All operations were performed under strict sterile conditions in a conventional operation room. The rats were positioned supine and the intercondylar notch region of the knee was palpated and identified. Using a percutaneous stab incision, a 20-G followed by 18-G and 16-G needles was inserted into the femoral canal through the intercondylar notch and advanced by hand reaming to a depth of 3.5 cm. The medullary cavity was then carefully inoculated with 5 μl of the different bacterial suspensions using a microsyringe. After the entire volume of the bacterial suspension was injected into the canal, the needle was slowly withdrawn. A UV-irradiated Ti6Al4V pin was press-fit into the canal by retrograde insertion. The control pin was inserted into the contralateral femur. The implants were fully inserted into the canal so that they did not interfere with the full range of knee motion (Fig. 2). The stab incision was then closed. Systemic antibiotics were not administered to animals at any time. Animals were sacrificed at different times between 24 and 72 h with an intracardiac injection of ClK (0.005 ml/g). The femur was disarticulated at the hip and removed in toto. The soft tissues were then stripped away from the femur and the implants retrieved.

### Sample analysis after extraction

Immediately after extraction, the samples (pins and femur) were immersed in sterile PBS. In order to remove the adhered bacteria, samples were first agitated with avortex for 30 s to separate the loosely bound microorganisms. Then they were removed from this liquid and again immersed in fresh sterile PBS for sonication in an ultrasonic bath (Ultrasons P Selecta, Madrid, Spain) for 15 min. This procedure ensured the detachment of tightly bound bacteria. After that, the PBS-containing bacteria were subjected to the serial dilution method in order to determine the total amount of bacteria in the solution. The agar plates were incubated for 18–24 h at 37 °C and the CFU were counted using a magnifying glass.

### Statistics

Data from contact angle experiments were analyzed using mean values and standard deviations. The distribution data for each sample was Gaussian. The statistical analyses in the animal experiments were performed using a software package (SPSS for Windows, release 15.0; SPSS, Chicago, IL, USA). The distribution score for each variable was checked using Kolmogorov–Smirnov and Shapiro–Wilk test. As the scores of each variable were not evenly distributed we used the non-parametric paired samples technique (Wilcoxon signed-ranks test). All *P* values <0.05 indicated significant differences between samples.

## Results

**Fig. 3:**
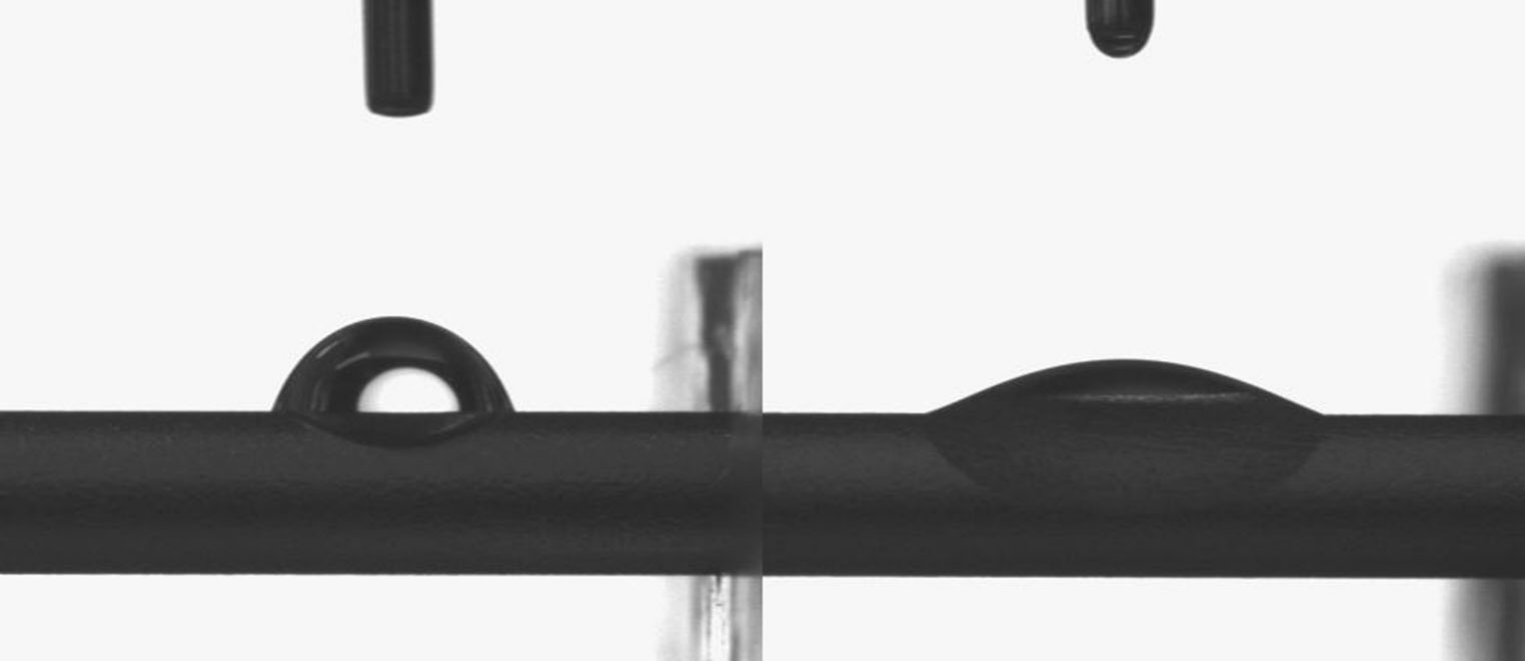
Water drops on a control pin (*left*) and irradiated pin (*right*)

Figure 3 presents two typical images of the water droplets on Ti6Al4V pins before irradiation (left) and after irradiation (right). An irradiation time of 48 h guaranteed complete hydrophilization of the surface of the pins. At this time, contact angles changed from 70º ± 3º to 12º ± 2º, similar to Ti6Al4V disks after 15 h of irradiation [27].

Of the 36 rats that had undergone surgery, two died within 3–5 h following surgery as a result of a failure at anesthesia induction without evidence of sepsis; the remaining 34 rats were included in the statistical analysis.

**Fig. 4:**
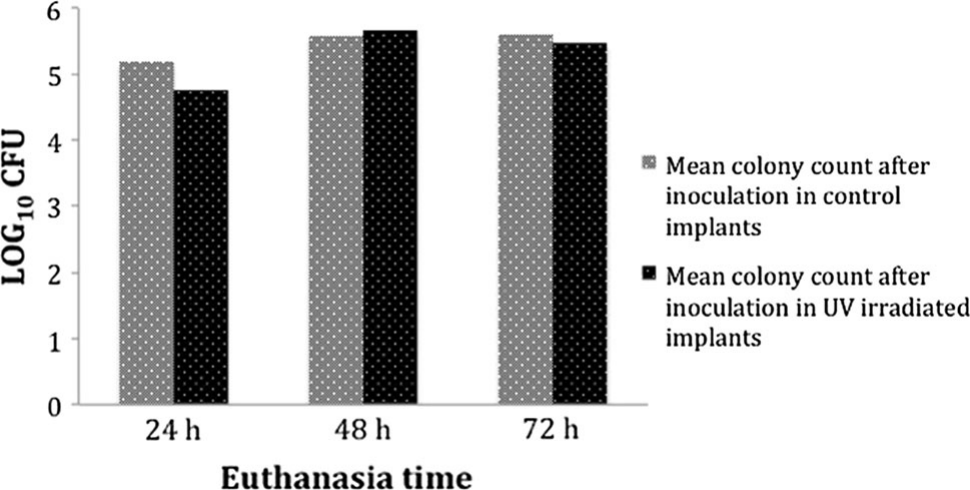
Mean values of bacteria harvested from irradiated pins against those collected from control pins (non-irradiated) for sacrifice times between 24 and 72 h

**Fig. 5:**
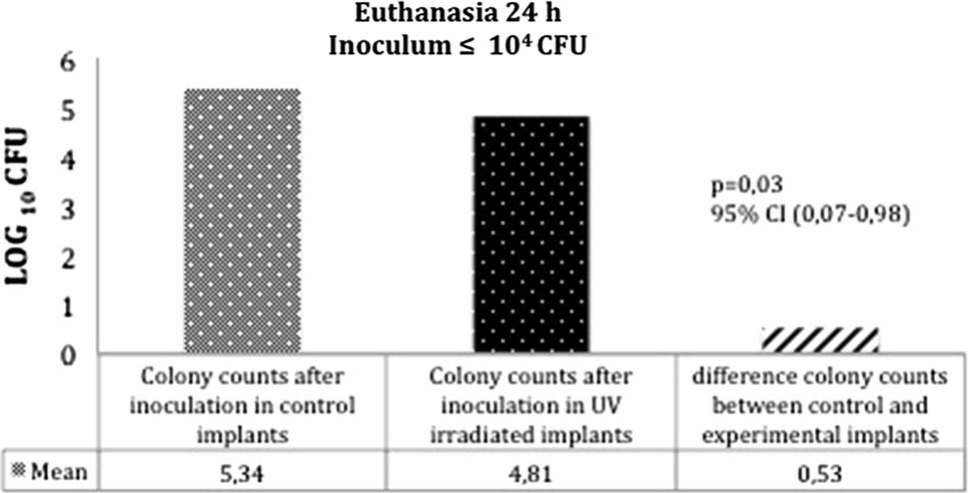
Statistically significant reductions (*P* < 0.05) in the mean values of bacteria harvested from irradiated pins against those collected from control pins (non-irradiated) for sacrifice times of 24 h and inoculums <10^4^ CFU

Tables 1, 2 and Fig. 4 show the summary of the mean number of CFU in the retrieved samples. On comparing paired samples (control and irradiated for implants and femurs) there were no significant differences in mean colony counts after inoculation between the control and experimental femurs at 24 and 48 h. At 72 h, the mean colony counts after inoculation in experimental femurs were significantly lower than those of the control group; the reduction percentage in the total number of bacteria adhered to the femur being 76 % (*p* = 0.041). A similar difference between control and experimental implants was observed at 24 h using inoculum doses <10^4^ CFU; the mean colony counts after inoculation in the UV-irradiated implant were significantly lower than those of the control implant with the reduction percentage being 70.48 % (*p* = 0.017) (Fig. 5).

**Table 1:** Mean of the total number of CFU detached from the femurs surrounding the irradiated pins ($\mathop {\overline{x} }$ Exp) against those surrounding the control pins ($\mathop {\overline{x} }$ Control) at 24, 48 and 72 h for the different inoculums employed

Time (h)	*n*	Femur log_10_ (CFU)					
		$\mathop {\overline{x} }$ Control	$\mathop {\overline{x} }$ Exp	$\mathop {\overline{x} }$ Control − $\mathop {\overline{x} }$ Exp	Binomial exact differences (95 % CI)		*P* Wilcoxon test
					Low	Upp	
24	11	5.21	5.17	0.04	−0.23	0.32	0.929
48	8	5.70	5.64	0.05	−0.49	0.60	0.575
72	15	5.89	5.26	**0.63**	0.03	1.23	**0.041**

Bold values indicate significant differences

*Upp* upper

**Table 2:** Mean of the total number of CFU detached from the irradiated pins ($\mathop {\overline{x} }$ Exp) and non-irradiated pins ($\mathop {\overline{x} }$ control) at 24, 48 and 72 h for the different inoculums employed

Time (h)	*n*	Implant log_10_ (CFU)					
		$\mathop {\overline{x} }$ Control	$\mathop {\overline{x} }$ Exp.	$\mathop {\overline{x} }$ Control − $\mathop {\overline{x} }$ Exp	Binomial exact differences (95 % CI)		*P* Wilcoxon test
					Low	Upp	
24	11	5.19	4.75	**0.44**	−0.02	0.90	**0.05**
48	8	5.57	5.77	−0.19	−0.54	0.16	0.263
72	15	5.59	5.46	0.13	−0.37	0.62	0.496

Bold values indicate significant differences

*Upp* upper

Comparison of paired samples (control and irradiated) showed statistically significant reductions (4630 %) (*P* = 0.046) in the median values of the total bacteria harvested from the pins and femurs; irradiated pins plus their surrounding femur versus non-irradiated pins plus their surrounding femur for inoculums <10^4^ CFU.

## Discussion

Despite the specific protocols for surgical asepsis, including implant sterilization, disinfection of the surgical area and appropriate antibiotic prophylaxis, total elimination of bacterial contamination is impossible and infection incidence related to surgical implants ranges from 0.2−17.3 % [29–31]. The first step in the development of an infectious process is microbial adhesion to the surface of the implant [32]. This step, also called unspecific or reversible adhesion, seems to be mediated by physicochemical forces where the hydrophobicity of the substratum surface plays an important role. It is difficult to quantify the duration of this initial phase; some in vitro studies deal with the first minutes and others deal with several hours after the beginning of the experiments [33, 34].

Our research group has proposed UV irradiation as a simple, economic and efficient method to modify the physicochemical surface properties of Ti6Al4V [14]. Moreover, in vitro adhesion experiments with irradiated samples have shown that bacterial adhesion is reduced in relation to non-irradiated samples while the excellent response of the surface toosteoblast adhesiveness and proliferation is maintained [27].

UV light pretreatment of titanium surfaces markedly increases their osteoconductive capacity. New bone formation occurs extensively in UV-treated implants maximizing bone–implant contact by up to nearly 100 % at week 4 of healing; UV treatment enhances the strength of bone–titanium integration by >3 times at week 2 of healing [35].

In addition to physicochemical changes, we also observed an in vitro bactericidal effect on the Ti6Al4V surface for at least 1 h after turning off the UV irradiation source. This residual post-radiation effect can be crucial for minimizing early contamination of implants in an in vivo situation [28] and to avoid the harmful effects of UV radiation on humans. It would no longer be necessary to irradiate the material once implanted in the patient, but only prior to implantation.

Before the samples were analyzed, the experimental animal model was rigorously studied and tested for its effectiveness in reproducing an early infection scenario. Different osteomyelitis experimental models have been proposed in the bibliography using a variety of strategies and agents to induce osseous infection. As an example, authors used morrhuate sodium or its derivative arachidonic acid to induce aseptic osseous necrosis, which is an ideal situation for bacterial proliferation [36]. The insertion of foreign bodies provides a surface for bacterial adhesion and further biofilm formation, resulting in an increase in bacterial resistance to the host defenses [37]. The bibliography also shows studies with different materials—osseous cement [38], osteosynthetic materials such as needles and nails [39], and osseous wax [40]. This latter substance and osseous cement are also employed to seal the hole created in the bone to avoid the overflow of the inoculum and the formation of abscesses in surrounding soft tissues. Other authors have induced thermal osseous necrosis by electrocauterization or by using drills to open the intramedullar channel of the bone [41]. Our animal model avoided the use of sclerosing agents, drilling to open the femur channel and osseous wax to seal the femur hole, with the objective of isolating the effect of the titanium surface as a foreign body to induce osteomyelitis.

In conjunction with the experimental model proposed, the possibility of contamination during the collection of samples was also examined and always ruled out. Nevertheless, variability in the bacterial counts was always displayed, as indicated in Tables 1 and 2, reflecting the typical dispersions of an in vivo model, i.e., anatomic changes in the rat femurs, variations in the milling process or in the collocation of the implants, bleeding in the femoral canal and the specific characteristics of the rat immunological system.

In addition to selecting a suitable animal model to detect differences between non-irradiated and irradiated samples, we were also aware of the importance of selecting an appropriate inoculum range to observe changes. In this case, the selection was based on those ranges found in the bibliography and our own results as the investigation progressed. Darouiche et al. performed a pilot trial to compare the ability of different inoculums of *S.aureus* to cause infection in rabbits and concluded that a bacterial inoculum of 5 × 10^2^ CFU was optimal as it resulted in colonization of most of the Ti6Al4V pins inserted in the medullary canal of the femur [42]. Akiyama et al. presented the antibacterial activity of silver-containing hydroxyapatite by inoculating the medullary cavity of rat tibia with 1.0 × 10^2^ CFU [43]. Melcher et al. carried out an interesting study on the establishment of an animal model to permit investigation of the role played by the hollow nail design in the pathogenesis of intramedullary infection, and their bacterial inoculums varied from 2 × 10^3^ to 4 × 10^7^ CFU [44]. In some osteomyelitis models the number of bacteria used in the inoculated biofilms was as high as 10^8^ [45]. Monzon et al. indicated that the use of ‘only’ 10^6^ bacteria in biofilm was sufficient to guarantee osteomyelitis infection [46]. In the work of Schlegel and Perren on surgical aspects of infection involving osteosynthesis implants the number of colony-forming staphylococci varied from 2 × 10^3^ to 4 × 10^6^ [47]. With this information the research has focused on ‘low’ (10^3^ CFU), ‘intermediate’ (in the order of 10^4^ CFU) and ‘high’ (in the order of 10^6^ CFU) inoculums. The number of cases of low inoculums was higher than the others because this situation is closer to reality, as we will discuss further. The main objective in this study was to demonstrate the in vivo bactericidal effect of UV irradiation on Ti6Al4V surfaces. Two types of variable were studied—time elapsed since UV irradiation and inoculum size.

For inoculums up to 10^4^ CFU, the mean reductions in the adhered bacteria of irradiated versus control implants were >70 % (Fig. 4). However, there were no reductions at the highest inoculum. In line with our results, Melcher et al. observed differences between solid and slotted nails, and demonstrated that for inoculums of ≥4 × 10^5^ CFU, the nail design had no effect on susceptibility of infection since the total infection rate for this level of inoculation was too high [44].

Our results enhance the effectiveness of the treatment at the initial stage of the infectious process. Other in vivo works have also selected similarly short observation−harvest times for studying specific treatments in animals. For example, Fujimura et al. investigated the effect of different antibiotics (alone or combined) on the eradication of *S. aureus* biofilms on titanium devices and concluded that 72 h was the baseline time for the eradication of the biofilm using a combination of clarithromycin, cefazolin and vancomycin [48]. Zaizuhana et al. tested a traditional herbal medicine used in a Malay community using observation−harvest time points of 24, 48 and 72 h [49]. Akiyama et al. recently developed a novel antibacterial coating of hydroxyapatite, and bacterial counts were calculated for rats sacrificed at 24, 48 and 72 h postoperatively [43].

The reductions observed not only in the prosthesis but also in the femur are in agreement with the antimicrobial mechanisms we propose for the post-irradiated surfaces. On the basis of the absence of photocatalytic reactions on the irradiated surfaces after turning off the UV lamp, the changes observed can be attributed to the hydrophilization of the irradiated surface and the combined effect of surface radiation emission and surface conduction with the recombination of the electron–hole pairs generated in the TiO_2_ semiconductor during the irradiation process [28, 50]. Hydrophilization affects the bacteria in intimate contact with the implants and leads to a weaker bond between the microorganisms and the substrate. However, radiation emission and surface conduction on the excited Ti6Al4V surface must influence not only the bacteria close to the prosthesis but also those located at longer distances, as demonstrated from the important bacterial reductions observed in the femur. This behavior had been previously predicted through in vitro experiences by studying the viability loss of bacteria deposited not directly on the irradiated surfaces but on thick films which had been deposited on the irradiated Ti6Al4V samples [28].

Our in vivo study does not represent a real clinical situation but rather the worst scenario, i.e., the implant is suddenly infected with an important bacterial inoculum and there is no antibiotic prophylaxis. In clinical medicine this antibiotic prophylaxis is standardized and early bacterial contamination is due to an accident and/or the surgical process, which is lower than in any of the studied cases. It is recommended for conventional operating rooms that the bioload should not exceed 35 CFU m^−3^ in an empty room or 180 CFU m^−3^ during an operation [51, 52]. In this context, the relatively short-lived activity of irradiated Ti6Al4V is intended to effectively combat bacteria that are introduced perioperatively into the implantation site as a result of contamination of the implant, bone or tissue and for this reason the irradiation of the prosthesis prior to the implantation process could be even more effective than in the in vivo situations presented in this work.

In conclusion, the irradiation of Ti6Al4V implants with UV-C light prior to the implantation in rats reduces bacterial colonization on the material surface during the first 72 h after surgery. This bactericidal effect is also observed in the surrounding femur due to the long-distance activity of the excited surface, as shown in previous in vitro experiments. Although the results of this animal study demonstrate the protective efficacy against early infections of this antibiotic-free treatment, the potential clinical benefit of irradiated devices can only be confirmed in large human trials.
